# Rancid rumors or Native wisdom: Evaluating the efficacy of animal fats as insect repellents attributed to historic-period Native Americans

**DOI:** 10.1371/journal.pone.0301677

**Published:** 2024-07-17

**Authors:** Delaram Esmaeili, Keyla R. Salas, Hailey A. Luker, Soumi Mitra, Claudia J. Galvan, F. Omar Holguin, Sophie Whyms, Immo A. Hansen, August G. Costa

**Affiliations:** 1 Department of Biology, New Mexico State University, Las Cruces, NM, United States of America; 2 Department of Neuroscience, Baylor College of Medicine, Houston, TX, United States of America; 3 Department of Plant and Environmental Sciences, New Mexico State University, Las Cruces, NM, United States of America; 4 Department of Anthropology, Rice University, Houston, TX, United States of America; 5 School of Pharmacy and Pharmaceutical Sciences, Trinity College, Dublin, Ireland; Universidade Federal do Para, BRAZIL

## Abstract

Little is known about Native American adaptations to blood-sucking arthropods prior to and following European contact. Multiple accounts starting in the 16^th^ century suggest that rancid animal grease was employed by Gulf Coast indigenes as a mosquito repellent. Although many Native American ethnobotanical remedies for biting insects have been recorded, the use of animal products for this purpose is not well documented. Moreover, few traditional Native American mosquito repellents have been examined using controlled laboratory methods for repellency testing. In this study, we tested the repellent efficacy of fats derived from alligator, bear, cod, and shark that were aged to various stages of rancidity. Using yellow fever mosquitoes, (*Aedes aegypti*), we performed an arm-in-cage assay to measure the complete protection times resulted from these fats, when applied to human skin. We used a Y-tube olfactometer assay to evaluate long-distance repellency and tested tick-repellency in a crawling assay. Our results suggest that rancid animal fats from cod, bear, and alligator are potent albeit short-lived mosquito repellents. We found that both rancid and fresh fats do not repel ticks. Our findings show the validity of traditional ethnozoological knowledge of Native American people and support aspects of the ethnohistorical record.

## Introduction

Blood-sucking insects from the mosquito family (*Culicidae*) have been a major nuisance and deadly threat throughout human history [1]. Mosquitos are pests that seek out their hosts and leave irritating bites. More importantly, mosquitos are vectors for harmful and deadly disease-causing pathogens that cause hundreds of thousands of human deaths annually [2, 3]. As such, mosquitos have had a massive influence on human development and a wide range of human adaptations to these deadly pests have been documented throughout history [4, 5]. These include cultural adaptations such as avoidance through migration, the use of repellent substances and spatial repellents like dense smoke, the construction of protective barriers like clothing and netting, and more recently, the use of various more advanced mosquito control measures ranging from the use of insecticides to mosquito population control strategies using transgenic technology [6–8]. In addition to mosquitoes influencing human behavior culturally, the diseases they transmit have also influenced humans genetically. In the Old World, *Anopheles* mosquitos that transmit malaria parasites (*Plasmodium spec*.) have driven the selection for numerous distinct genetic adaptations in human populations within the past 7,000 years. Examples of these adaptations are the sickle cell trait, glucose-6-phosphate deficiency, thalassemia, and Duffy-negative blood groups [9, 10]. Due to the selective pressure mosquito-borne diseases provide, mosquitos are considered to be one of the strongest drivers for evolutionary selection on the human genome in recent history [11]. To this day, despite many innovations for mosquito population control, mosquitos continue to impede human activities and influence human behavior [12].

While the impact of mosquitos on human history in the Old World is well documented, there are severe gaps in knowledge on this topic in the New World [1, 5]. The dynamic of interactions between Indigenous Americans and mosquitos, both prior to and following European contact, has remained understudied. Some questions that have yet to be answered are: How and to what extent did mosquitos influence the behaviors of Indigenous Americans? What methods did Native Americans use to deal with mosquitos? What impacts did the introduction of invasive Old World mosquito species such as the highly anthropophilic yellow fever mosquito, *Aedes aegypti*, have on Indigenous Americans?

The coastal bend of Texas and adjacent states host some of the densest mosquito populations in the United States [13]. Warm weather and the abundance of standing water makes this region a prime mosquito habitat. Prior to major mosquito control efforts in the 1920s, mosquito populations were unabated in this region which was correlated with rampant and frequent outbreaks of diseases like dengue and yellow fever [14, 15]. There are reports of yellow fever and dengue outbreaks in the port town of Galveston, TX along with isolated settlements in the Rio Grande Valley. Between 1830 and 1870, 4,279 people in this region died from yellow fever outbreaks [16, 17]. In 1922, an outbreak of dengue killed nearly 30,000 people [1, 18].

Historical documents from Spanish and French expeditioners in the 1500s and 1600s provide insights into the impact mosquitos had on Indigenous Americans (see **Table 1**). Mosquitos appear prominently in the accounts from the survivors of the 1527 Spanish Narváez expedition. The Narváez expedition survivors who were shipwrecked along the coast of what would later become Texas, reported that the mosquitos in this region were deadly, especially for people who wore little clothing. One of these shipwreck survivors, Álvar Núñez Cabeza de Vaca, reported becoming a captive of coastal indigenes, and described being forced to tend the smudge fires that were used to repel mosquitos at night [19]. Another account that mentioned mosquitos within the New World was from the Frenchman Henri Joutel. During the 1684–1687 La Salle Expedition to Texas, Joutel described a maddening number of mosquitos which made sleep impossible. Joutel noted some mosquito control methods that were used within the Gulf Coast area. These methods included smudging and setting grass fires to create smoke for repelling mosquitos and to destroy mosquito habitats, and the construction of elevated shelters designed to leverage wind to deter mosquitos [20]. Another Frenchman, Simar Francois De Bellisle, who was marooned with the Akokisa peoples of the Galveston Bay area in 1719 remarked that “there were so many mosquitos that I thought I would die" [21] and went on to spend a night submerged in water rather than being exposed to the onslaught.

**Table 1:** Historical accounts.

Source	Source and Observation Date	People	Location	Animal Fat Source	Quote
Dumont (1753:140–141), Swanton 1911	Primary 1715–1747	Natchez	Natchez, Mississippi Region	Black Bear	“They have the custom of rubbing themselves frequently with bear’s oil, which protect[s] them from the bites of mosquitos”
Du Pratz (1774:308)	Primary 1718–1734	Natchez	Natchez, Mississippi Region	Black Bear	“they rub them [their skin] with bear’s oil to prevent the flies from biting them”
Du Pratz (1774:332–333)	Primary 1718–1734	Choctaw		Unknown	the fat with which they rub their skin and their hair, and to their manner of defending themselves against the moskitos, which they keep off by lighting fires and standing in the smoke
Alice Oliver in Gatschet (1891:81)	Primary 1830s	Karankawa	Matagorda Bay Region Texas	Shark liver oil	“the odor of the shark’s oil with which they habitually anointed their entire bodies as protection against mosquitoes, rendered them very offensive”
Noah Smithwick (1900:13)	Primary 1820s	Karankawa	Lower Lavaca River Texas	Alligator	“their ugly faces were rendered hideous by the alligator grease and dirt with which they were besmeared from head to foot as a defense against mosquitos”
Dyer (1917:5)	Secondary 1817–1820	Akokisa and Atakapa	Southeast Texas and Southwest Louisiana	Alligator, fish	Alligator “oil served as a delicacy, and placed in jugs for future uses; an essential one was for body inunction, which kept off mosquitoes……The smell was abominable”
Newcomb (1961:324)	Secondary	Akokisa and Atakapa	Southeast Texas and Southwest Louisiana	Alligator	“Oil obtained from these reptiles [alligator] was used on the skin to repel mosquitoes, a practice often cited as the chief cause of the coastal natives’ repulsive odor”
Carr (2010:33)	Secondary 1804	Lewis and Clark Expedition	Missouri River Valley	Beef Tallow and Pig Lard	He [Lewis] thoughtfully acquired 11 mosquito curtains and ensured an ample supply of hog’s lard. When smeared on the skin, the lard was supposed to ward off mosquitoes

**Fig 1:**
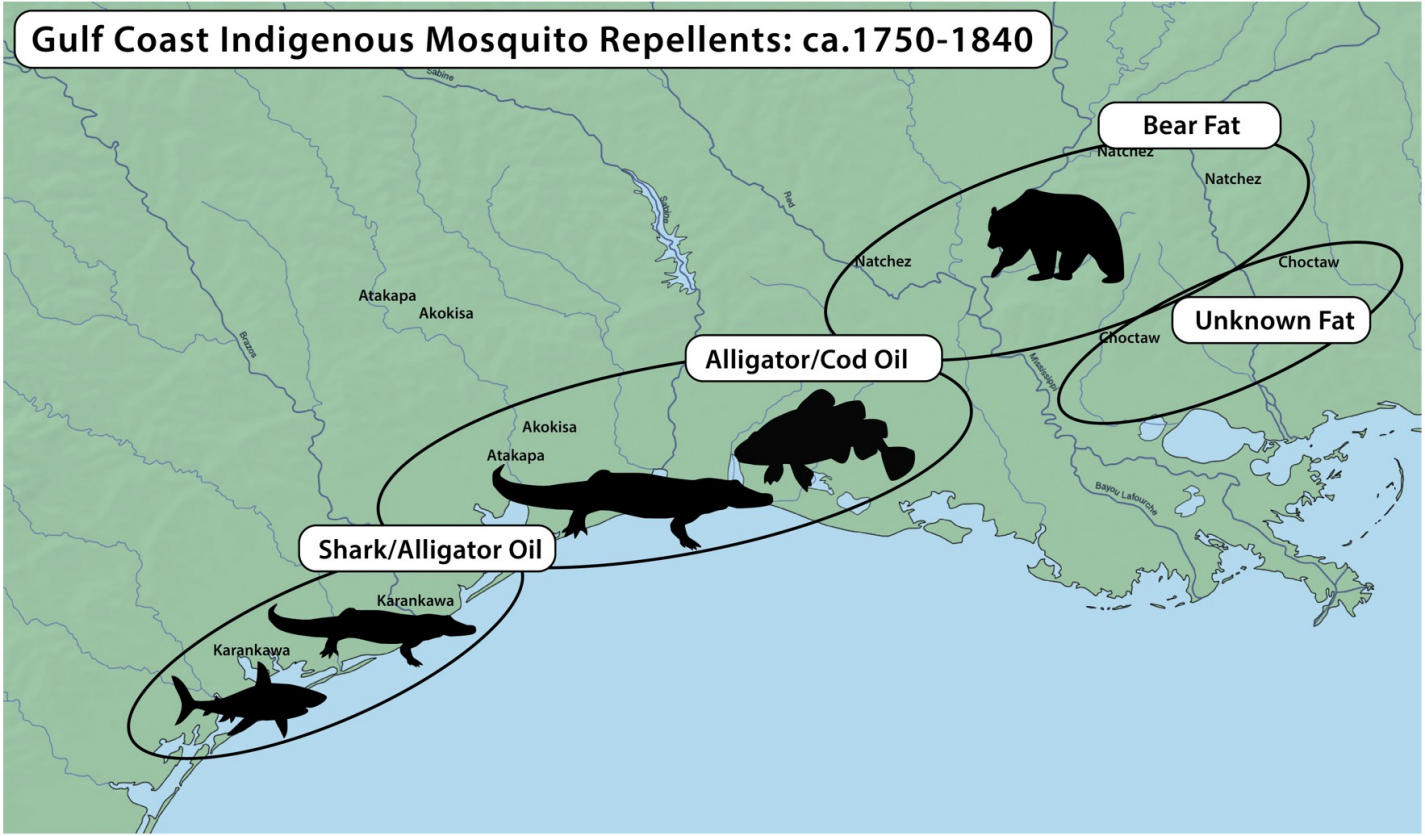
Ethnohistorically reported use of animal fats as insect repellents. Shown are regions within in the Gulf of Mexico that have historically documented use of specific animal fats as repellents. This image was generated using Adobe Illustrator 25.4.1 https://www.adobe.com/products/illustrator.html. The base map was generated by QGIS 3.34.3-Prizren https://qgis.org/en/site/ using raster and physical data from Natural Earth https://www.naturalearthdata.com/downloads/. Note: This map does not represent or intend to represent official or legal boundaries of Indigenous nations.

There are limited ethnohistorical accounts before the 1850s that discuss local mosquito control measures in the New World. Several primary and secondary accounts have been identified from people who traveled the Gulf Coast between 1750 and 1820 (see **Table 1**). These accounts suggest that Indigenous Americans anointed themselves with pungent rancid animal fat, that was either oil or grease, to repel mosquitos (see **Fig 1**). The European travelers of this time described being repulsed by the scent of these mosquito repellents and wrote prejudiced descriptions about Native American groups on this subject. In the 1700s, French travelers reported that the Natchez people of the lower Mississippi Valley utilized black bear (*Ursus americanus*) grease as a mosquito repellent, sunblock, and multipurpose ointment [22–24]. The Choctaw were also reported to have used a type of animal fat as a mosquito repellent, which Antoine Simon Le Page Du Pratz [23] found olfactorily offensive. In the early 1800s, the Karankawa peoples of the Texas coastal bend, were documented to have used alligator or shark oil (presumably shark liver oil from some unknown shark species) as a mosquito repellent. Around the same time, the western Atakapa and Akokisa people that lived along the coastal homelands of what are now southeast Texas and southwest Louisiana, were documented to use either alligator (*Alligator mississippiensis*) oil or an unspecified fish oil as mosquito repellents [25, 26].

Although many Native American ethnobotanical remedies for biting insects have been recorded such as goldenseal (*Hydrastis canadensis*), western yarrow (*Achillea millefolium L*. *var*. *occidentalis DC*) and sweetgrass (*Matricaria discoidea*) [27], the use of animal products for this purpose is scarcely considered in the literature. Native Americans across the New World have long used traditional knowledge in making use of animal fats as insect repellents and for other purposes. Notably, bear grease serves as a key ingredient in various forms of insect repellents and personal ointments, especially amongst Iroquoian-speaking people [28]. A search of the Native American Ethnobotany Database (NAEB) yielded 37 botanical items documented for use as repellents; of these seven (mostly hickory and oak nuts) are said to have been combined with bear grease by Indigenous Americans [29]. Many other tribes including the Delaware, Ojibwa, Kutenai, and Comanche have utilized bear fat for multiple purposes [30]. However, the effectiveness of animal fats as mosquito repellents is not completely known.

Based on above mentioned historical accounts, we hypothesize that rancid animal fats repel biting insects, specifically mosquitoes. In this paper we investigate the efficacy of four different types of animal fat (bear, alligator, shark, and cod) as mosquito and tick repellents. Fats were aged to varying degrees of rancidification by long-term exposure to air and sunlight and subsequently tested in spatial- and contact repellency assays [31]. Finally, Gas Chromatography/ Mass Spectrometry (GC-MS) analysis was performed on the samples to assess the odorant composition of these repellents.

## Materials & methods

### Fat Sample acquisition and rancidification

**Fig 2:**
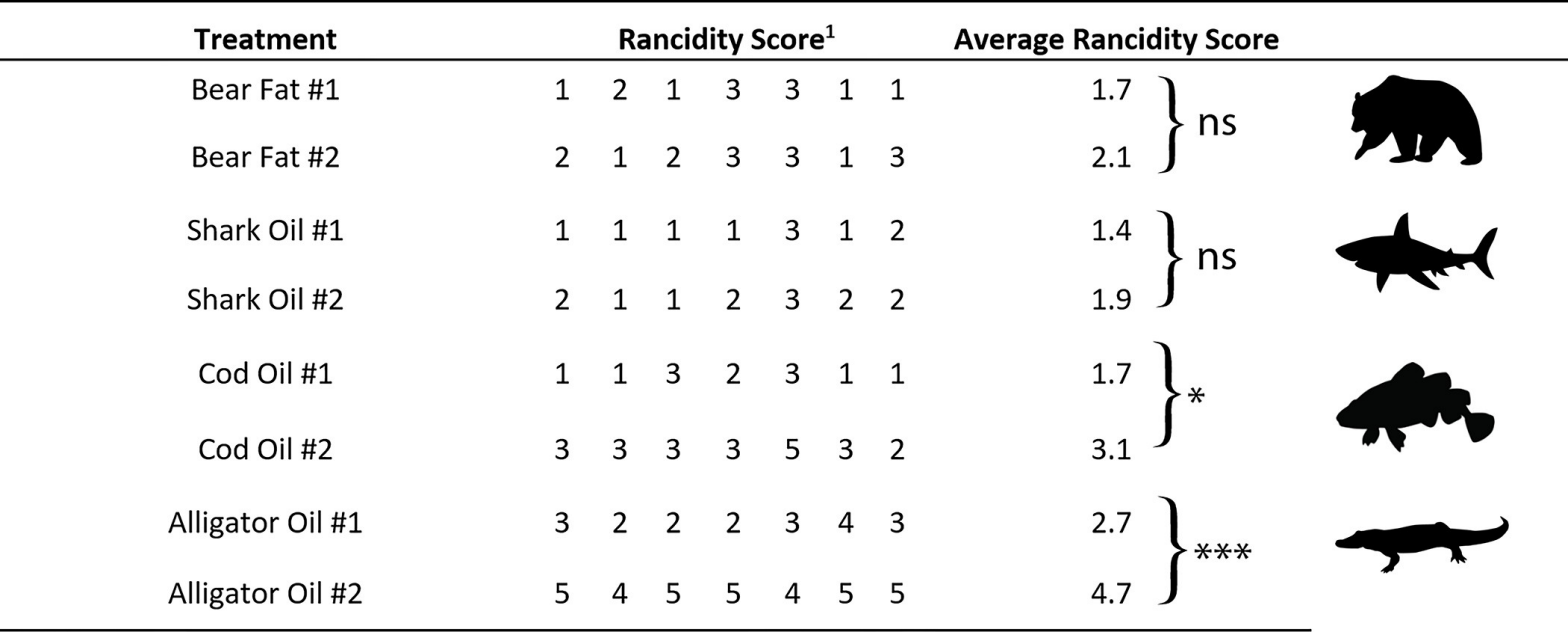
Rancidity scores for animal fats used in this study. ^1^Rancidity scores determined by human olfaction to rank fats on a scale from 1 to 5, with 5 being the most pungent smell. The asterisks denote significant differences between the different samples of each animal fat. (p ≤ 0.05*; p ≤ 0.001***; ns = not significant).

Eight paired samples from four animal species of contrasting levels of rancidity to freshness were acquired and prepared for testing. These animal fats were gathered from a variety of sources (**Table 2**). We are confident, that bear and alligator fat were isolated from the same species as the ones mentioned in the historical accounts. We cannot claim this for the shark oil. Spiny dogfish is a common shark in coastal areas, worldwide, but we lack information about species distribution in the historic fishing harvests of Gulf Coast Native Americans. Fresh animal fat samples were kept in cool and sealed storage. Animal fat samples were allowed to sit in subaerially exposed conditions (in direct sunlight) for between one to over three months to allow rancidification from oxidation and hydrolysis. The rancidification of fats occurs when they are exposed to oxygen, which results in the long-chain fatty acids to be broken into smaller compounds. The strong, pungent smell that is associated with rancid fats is due to the reactive molecules that are produced when the fat samples go through oxidation [32]. The rancidification state of each sample was not directly quantified, instead fat samples were rank ordered from fresh to spoiled (i.e., rancid) by seven volunteers using scent as a proxy (**Fig 2**).

**Table 2:** Components of animal fats.

Treatment	Product Name	Source	Processing Method	Ingredients	Manufacturer and Distributer
*Black Bear Fat #1* *(Ursus americanus)*	Bear Grease	Bear Fat Tissue	Rendered	Pure Bear Fat	Centralia Fur & Hide Inc., Centralia, WA
*Black Bear Fat*^1^ *#2 (Ursus americanus)*	Bear Grease	Bear Fat Tissue	Rendered	Pure Bear Fat	DustySwampProvisions/Etsy, Hardwick, VT
*Shark Oil #1* *(Squalus sp*.*)*	Pure and Natural Shark Liver Oil	Shark Liver	Wet Pressing	Shark Liver Oil	HBOilsCenter/Etsy, Westchester, IL
*Shark Oil*^1^ *#2* *(Squalus sp*.*)*	GO Squalene	Shark Liver	Wet Pressing	Shark Liver Oil	GO Healthy, Wellington, New Zealand
*Cod Oil #1* *(Gadus sp*.*)*	100% Pure & Liver Oil	Cod Liver	Wet Pressing	Norwegian Cod Liver Oil, Vitamin A, Vitamin D3, Omega 3 fatty acids,	Velona/Etsy, Mount Prospect, IL
*Cod Oil*^1^ *#2* *(Gadus sp*.*)*	Carlson Wild Norwegian Cod Liver Oil	Cod Liver	Steam Extraction	Norwegian Cod Liver Oil, Omega 3 Fatty Acids, Vitamin A, Vitamin D, Vitamin E, Natural Lemon Flavor	J. R. Carlson Laboratories Arlington Heights, IL
*Alligator Oil*^1^ *#1 (Alligator mississippiensis)*	Alligator Oil	Alligator Fat Tissue	Rendered	Pure alligator oil	Porter’s Gator Processing, Anahuac, TX
*Alligator Oil #2 (Alligator mississippiensis)*	Pure Alligator Oil	Alligator Fat Tissue	Sun Rendered	Pure alligator oil	Southern Snares & Supply, Hortense, GA

^1^To facilitate the rancidification of the fats, they were set outdoors in direct sunlight for a duration of 1 to 3 months. All other fats were used for testing in the original form they were received from the manufacturers.

### Ethics declarations

All experiments conducted in this study have been reviewed and approved by the New Mexico State University Institutional Review Board. We confirm that we followed all guidelines mentioned in our current application—(22010) ‘Insect and Tick-Repellent Research’ expired 09/2023. The Environmental Protection Agency’s (EPA) protocol “OPPTS 810.3700; Insect repellents for human skin and outdoor premises.” was used as a guide to perform our arm-in-cage and tick skin-crawling assays [33]. All participants were given and signed an informed consent form starting in June of 2022 and ending in December of 2022. All participants gave written consent on printed consent forms, following the consent procedure approved by the IRB. Vulnerable persons (i.e., minors, pregnant and nursing women, prisoners, immune-compromised individuals, and people with severe reactions to mosquito bites) were excluded from this study. Participants were advised to avoid alcohol, tobacco, and any scented products at least 12 hours prior to this study.

All animal products used for this study were obtained from suppliers (see **Table 2**) that confirmed to us that these products were ethically sourced. Specifically, bear fat was obtained from black bears (*Ursus americanus*) in Canada following hunting regulations issued by the Government of Quebec. Shark liver oil was extracted from Southern Pacific spiny dogfish (*Squalus sp*.) and were collected following the guidelines of the Ministry for Primary Industries (MPI) of the country of New Zealand. Alligator oil was sourced from farm-raised alligators from Georgia under the provisions of the Official Code of Georgia (O.C.G.A). Cod liver oil was obtained from regulated US cod fisheries that follow the Code of Conduct for Responsible Fisheries (CCRF) and other guidelines of National Oceanic and Atmospheric Administration (NOAA).

### Rancidity score

We developed a new assay to rank rancidity using human volunteers similar to one mentioned by Laska and Teubner [34]. Rancidity scores were acquired by pipetting 100 μl of each fat onto a 4 cm x 4 cm filter paper. The filter paper was placed in a petri dish and volunteers were asked to smell the filter paper at a distance of 5 cm. Volunteers were then asked to rank the score of how rancid the fats were from 1 to 5, with 5 being the highest rancidity score. For each individual volunteer, there was a 24-hour period between testing the scent of each animal fat sample.

### Mosquito culture

The UGAL (University of Georgia Laboratory) strain of *Aedes aegypti* mosquito was used for all experiments. This strain was donated from Alexander Raikhel’s laboratory at the University of California, Riverside. Approximately 500 eggs were put in a 13” x 20” pan filled with approximately three liters of distilled water. Cat food pellets (Special Kitty, Walmart Stores, Bentonville, AR) were added to the pan as food source for the larvae as they emerged. Once the pupae emerged after 2–3 days, they were sorted in small plastic cups filled with distilled water and kept inside Bug dorm insect cages (30x30x30 centimeters, Bug dorm Company, Taichung, Taiwan). A small glass flask containing 20% sucrose solution was also kept inside the cage with a cotton wick on the top of the flask as food source for adult mosquitos’ post emergence. The cages were stored in the insectary under 27°C temperature and 80% relative humidity. The approximate age of all the mosquitoes used in the experiments was ten to fourteen days post-emergence.

### Ticks

Adult female *Ixodes scapularis* were used for all tick experiments. *I*. *scapularis* were acquired from the Oklahoma State University’s (OSU) Tick Rearing Facility. Once the ticks arrived, sharp forceps were used to poke two holes into the lid of the container they were shipped in. The ticks were kept hydrated by keeping a damp piece of paper in this container. Ticks were stored in an insectary room with a 12/12 light/dark cycle, a temperature around 27°C and a relative humidity of around 80%.

### Tick skin-crawling assay

**Fig 3:**
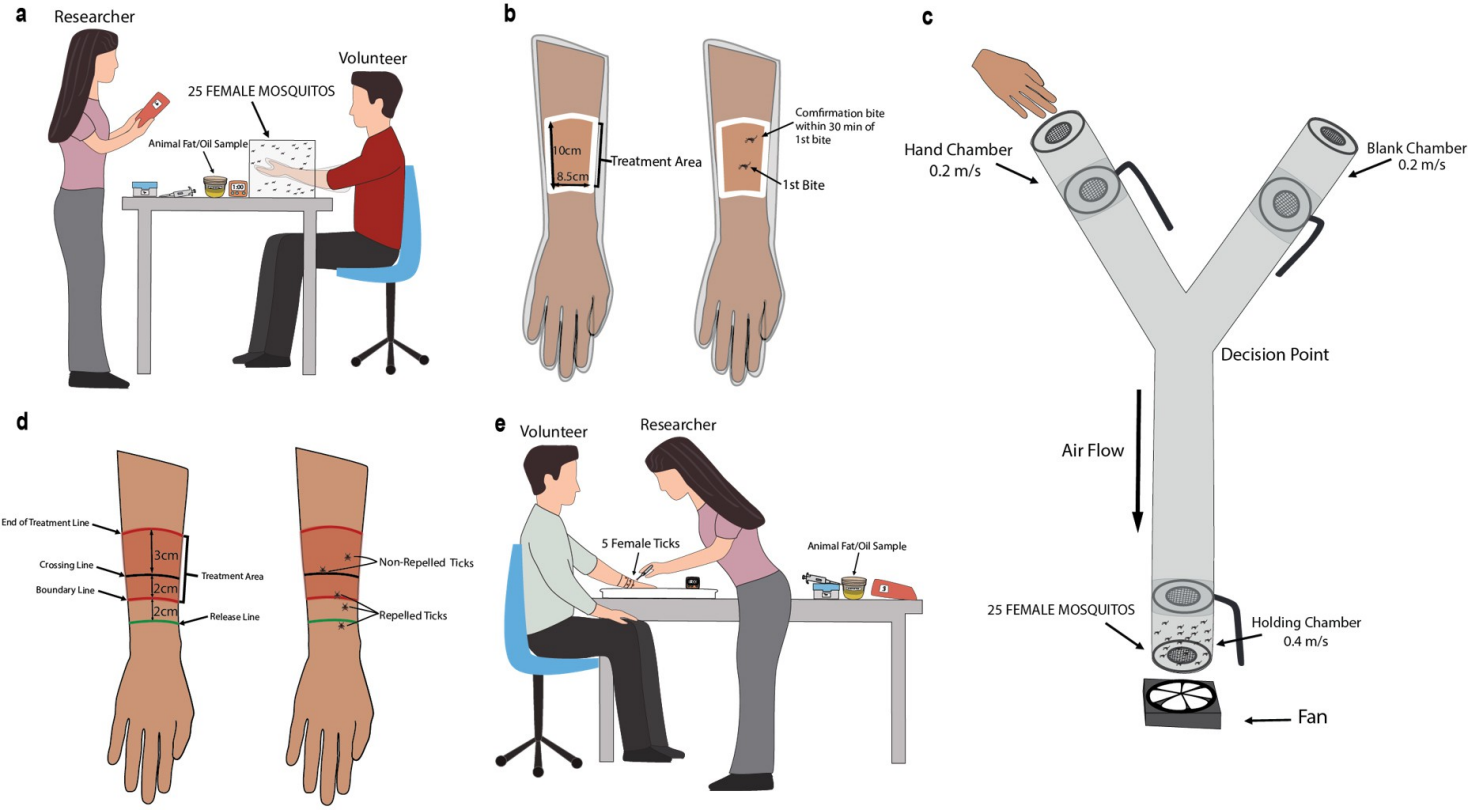
Setups of the assays used in this study. **a.** Arm-in-cage experimental setup. **b.** Dimensions of the treatment area and visualization of how CPT (complete protection time) was measured for the arm-in-cage experiment. **c.** Y-tube olfactometer experimental setup. **d.** Margin measurements of the experimental parameters for the tick skin-crawling assay. **e.** Tick skin-crawling assay experimental setup.

As previously described by Luker and coworkers [35], the tick skin-crawling assay (**Fig 3D and 3E**) was used to test the efficacy of animal fats as repellents. This assay was conducted based on the EPA protocol for testing insect repellants on human volunteers. Five female *I*. *scapularis* ticks were placed in a plastic cup with a lid over it prior to being used for the experiment. The volunteer was asked to wash their arms with unscented soap and dry it with a paper towel. With an unscented ballpoint pen, several lines were drawn on the volunteer’s arm. The first line was the release line, 2 cm before the boundary line was drawn. A third line was drawn 2 cm above the boundary line to indicate 2 cm into the treatment area and this line was referred to as the crossing line. The final line was drawn 5 cm above the boundary line to mark the end of the treatment area. After the lines were drawn, a control trial was conducted with no treatment to verify the questing behavior of the ticks. Volunteers were asked to place their arm in a 17 cm x 15 cm x 2 cm white pan with the palm of their hand facing up and their arm slightly angled up. Using featherweight forceps, five female *I*. *scapularis* ticks were then placed at the release line and would be permitted one minute to pass the crossing line. If this was accomplished, the ticks would qualify for testing of the different treatments. Testing of treatment was initiated by applying 250 microliters of the different fats to the treatment area in the volunteer’s arm. Once the treatment was applied, a timer was started. The tick crawling assay was done on volunteers for 5 minutes in 30-minute intervals until two ticks passed the crossing line. The second tick had to cross within 30 minutes of the first crossing. The time at which each tick passed the crossing line was recorded. The average of different replicates was calculated to determine the complete protection time.

### Y-tube olfactometer assay

In this experiment, a Y-tube olfactometer assay (**Fig 3C**), identical to the one described earlier [36, 37] was used to measure spatial repellency. The Y-tube assay was performed following the guidelines provided by the World Health Organization (WHO) in their “Guidelines for efficacy testing of spatial repellents” [38]. All the experiments were conducted between 0800 and 1200 hours on each day. Four volunteers were used for this experiment on different days. The volunteer used was not allowed to shower or use any personal hygiene products as that might impact the experiment. On the day of the experiment, around 175 microliters of fat was applied to the volunteer’s arm and placed adjacent to the “hand” chamber. Around 15–25, week old, overnight starved, female *Aedes aegypti* UGAL mosquitoes were released into the Y-tube “Holding” chamber. While airflow was adjusted at 0.2 meters/sec, mosquitoes in the “holding” chamber could sense the compound in the experiment. After thirty seconds elapsed, the door of the “holding” chamber was opened to allow the mosquitoes to decide which direction to fly towards, the “hand” chamber of the Y-tube that had the volunteer’s arm covered in treatment or the “blank” port without any odorant. After two minutes, all the doors were closed and total number of mosquitoes that flew into the “hand” chamber was counted. The same steps were performed three more times totaling to four replicates. Between replicates, the used mosquitoes were blown out of the Y-tube and fresh batch of mosquitoes were aspirated into the “holding” chamber. The hand chamber and blank chamber in the Y-tube assay were rotated to avoid positional bias. Each day, only one product was applied to the volunteer’s arm for testing as applying both the products on the same day might have negative effects on the result. Before applying the fat on the volunteer’s arm a control run was performed.

The percent attraction was calculated using the formula:

$$\frac{\#\;of\;mosquitoes\;in\;“hand”}{{Total\;\#\;of\;mosquitos}^{*}}\times 100=\%\;Attraction$$

*Total # of mosquitoes = # of mosquitos in the hand chamber + Y-tube area + blank chamber

### Arm-in-cage assay

As previously described by Luker and coworkers [35], the arm-in-cage assay (**Fig 3A and 3B**) was used to test the efficacy of animal fats as repellents. In this experiment, a 10 cm x 8.5 cm area on a volunteer’s forearm was applied with approximately 175 microliters of the fat with the help of gloved fingertips. The volunteer then wore a nitrile glove that had an opening cut into it in such a way that the area of the skin treated with test compound was exposed. The volunteer then inserted their hand into a 50 x 50 x 50 cm^3^ mosquito cage with transparent walls containing approximately 25 adult, female *Aedes aegypti* UGAL mosquitoes ten to fourteen days post-emergence. The hand was placed in the cage for 15 minutes. If no bite was observed the hand would be removed from the cage and placed back in at the 30 minute mark for 5 minutes. Testing was continued in 30 minute intervals until two bites were observed. The same experiment was performed three more times, in total four replicates. The sample size for the arm-in-cage repellency study consisted of 25 female mosquitoes for each replicate. There were four replicates that were completed with four different volunteers being used. The volunteers consisted of half males and half females with different ages and skin color. Between replicates, the used mosquitoes were aspirated out of the cage and a fresh batch of mosquitoes aspirated into the cage. For each replicate, if the volunteer got one bite, the replicate was considered as “Fail”. If the volunteer did not get any bite within the five minutes, then the replicate was considered “Pass”. After running 4 replicates, the percent protection efficiency (% PE) was calculated by averaging the number of pass and fail for each replicate. Animal fat was concluded to provide 100% protection efficiency if all the four replicates were “pass” i.e., the volunteer did not get a single bite in all the four replicates tested. If one of the four replicates was a “fail”, then the animal fat provided 75% protection efficiency.

### Gas Chromatography/Mass Spectrometry

Each fat was diluted in methylene chloride at a final concentration of 10% v/v. Diluted fats were analyzed by GC/MS using a Varian model 3400 GC with an RTX-5 column (30 m × 0.25 mm fused silica capillary, 0.25 μm film thickness), coupled to a Saturn 2000 ion trap mass spectrometer (EI, 70 eV). Helium carrier gas flowed at 1 mL/min, and injector and transfer line temperatures were 270 and 180°C, respectively. The initial column temperature was 50°C and held for 1 min, with a linear gradient of 15°C/min and held for 5 minutes with a total run time of 23.33 minutes.

Deconvolution and spectra processing was performed using Mass Spectrometry-Data independent Analysis software (MS-DIAL) [39]. Varian generated MS files were converted into net CDF format using OpenChrom [40] for data retrieval in MS-DIAL. Automatic peak detection was performed on peaks with a minimum peak amplitude of 500. For identification and alignment, we calculated Kovats retention index based on a C7-C40 saturated alkane mix Sigma Cat#49452-U and performed spectral matching with a cut-off of 80%, and calculated retention index tolerance of 50 to the Essential Oils GC/MS library [41]. Peak alignment was performed using a reference sample that contained equal amounts of each fat. Putatively identified compounds were assigned odor categories based on the olfactometry reports found at Flavornet https://www.flavornet.org/ and organoleptic properties found in The Good Scents Company Information System http://www.thegoodscentscompany.com/index.html. The resulting radar odorant charts are the visualization of the relative summed peak area in the representative odorant categories.

### Statistical analysis

All analyses were done using the program GraphPad Prism 10.1.2. A Shapiro-Wilk test was used to assess the normality of the data sets of each group. For normally-distributed data, we ran a one-way ANOVA followed by a Tukey’s multiple comparisons test to assess statistical significance between different groups. If the data was not normally distributed, a Kruskal-Wallis test with a Dunn’s multiple comparisons test was used to assess significance between different groups. A correlation analysis between rancidity scores and CPTs in the arm-in-cage was performed. First the average values of each measurement were calculated. The data was then analyzed for correlation by computing a Pearson correlation coefficient and a two-tailed p value. All other settings were left as default. A simple linear regression line was fitted onto the correlation analysis to display the relationship of the two variables. The above steps were repeated to analyze the correlation between rancidity score and percent attraction for the Y-Tube assay results.

## Results

### Historical evidence

**Table 1** provides the results of our literature research on animal fat use by Native Americans as mosquito repellents in the Gulf Coast region. We found five primary sources and three secondary sources ranging from 1715 to 1917 that suggest that different tribes within this region used animal fat repellents.

### Survey to rank the rancidity of fats

**Table 2** provides a description of the individual animal fats that were used in this study. Of the fats tested in this survey, Alligator Oil #2 had the highest rancidity score while Shark Oil #2 had the lowest rancidity score (shown in **Fig 2**). Overall, all rancid versions of a given animal fat scored a higher rancidity score compared to their fresh counterparts, except for Shark Oil #2 which was reported to be less rancid than Shark Oil #1. Alligator Oil #2 received an exceptionally strong rancidity score compared to the other oils.

### Gas Chromatography/ Mass Spectrometry (GC/MS) analysis of animal fats

**Fig 4:**
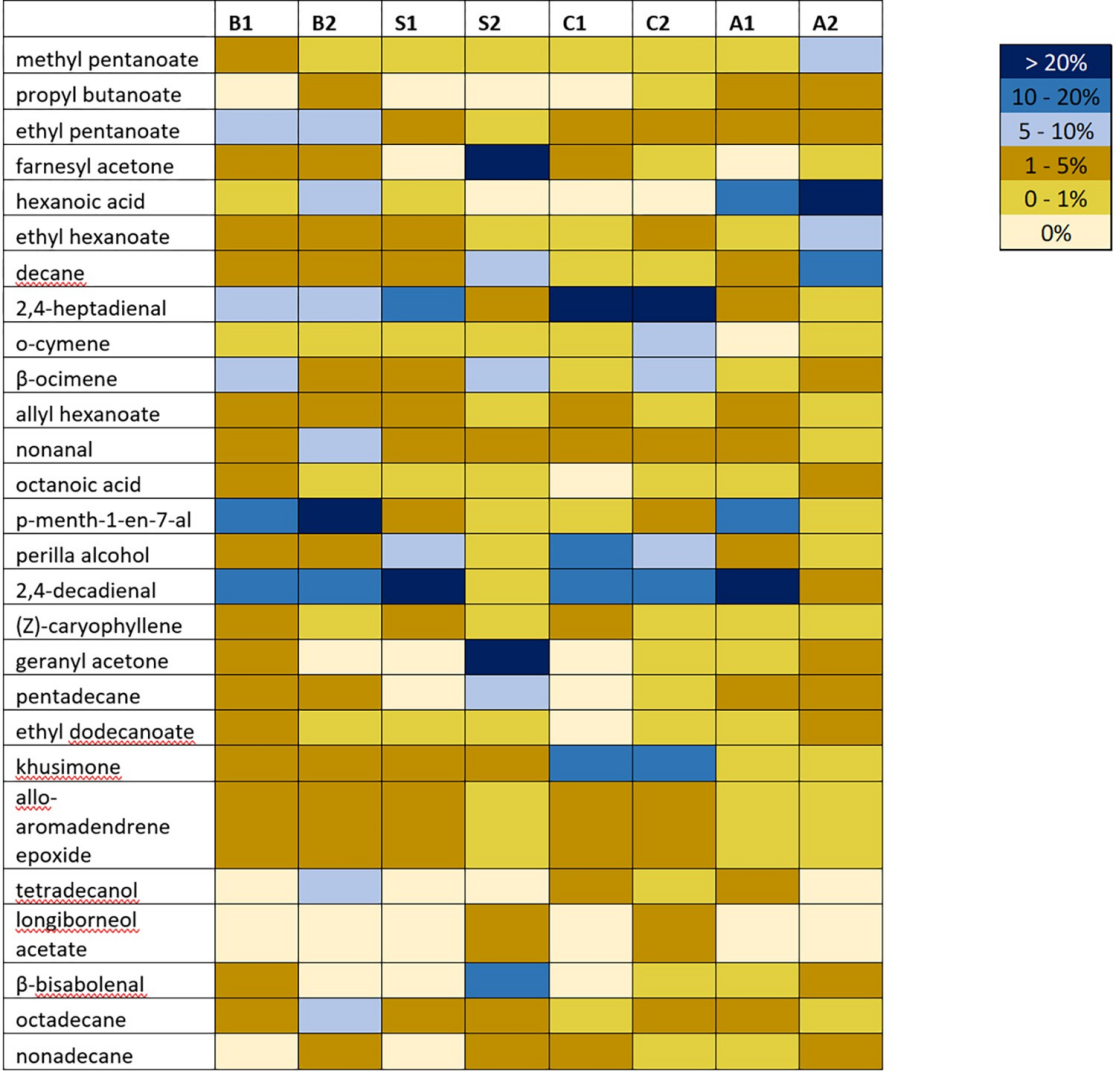
GC/MS odorant composition of fats used in this study. Shown are percentage concentrations for each identified compound, with 0% meaning the compound was not detected. The labels above columns two through nine indicate the different fat types; B1—Bear Fat #1, B2—Bear Fat #2, S1—Shark Oil #1, S2—Shark Oil #2, C1—Cod Oil #1, C2—Cod Oil #2, A1—Alligator Oil #1, A2—Alligator Oil #2.

A total of 27 components were identified across the eight samples of four animal fats, fresh and rancid (**Fig 4, Table 3**). 4 were monoterpenes, 7 esters, 4 alkane hydrocarbons, 3 sesquiterpenes, and 9 were other compounds such as ketones, carboxylic acids, and aldehydes (**Fig 4** and **S1 File**). Notable compounds include hexanoic acid (mean percentage concentration = 15.51%), 2,4 –decadienal (mean percentage concentration = 18.73%), and 2,4-heptadienal (mean percentage concentration = 11.79%). These carboxylic acids and/or aldehydes are common products of the rancidification of fats. Across all samples tested, monoterpenes, as a group, occurred at the greatest concentration (mean percentage concentration = 15.76%), with esters occurring at the second highest concentration (mean percentage concentration = 10.67%). Bear 1fat, shark oil, and cod oil had an increase in monoterpene content after rancidification. Additionally, an increase in alkane hydrocarbons is observed for rancid bear, shark, and alligator oil.

**Table 3:** Composition profiles of animal fats analyzed via GC/MS.

	R.I.	B1	B2	S1	S2	C1	C2	A1	A2	Compound Type
**menthyl pentanoate**	821	0.5	0.35	0.23	0.08	tr	tr	0.09	9.56	ES
**propyl butanoate**	898	-	3.73	-	-	-	0.69	3.15	1.53	ES
**ethyl pentanoate**	901	9.65	5.31	2.78	0.91	1.19	1.18	1.97	1.41	ES
**farnesyl acetone**	901	3.72	3.21	-	22.49	1.02	0.35	-	0.58	OC
**hexanoic acid**	967	0.97	7.18	0.65	-	-	-	16.69	52.06	OC
**ethyl hexanoate**	997	3.86	1.91	1.87	0.86	0.99	1.26	0.76	8.74	ES
**decane**	1000	3.86	3.99	1.87	7.53	0.99	0.97	1.42	10.38	AH
**2,4-heptadienal**	1005	5.26	5.24	13.85	1.29	33.33	30.3	4.39	0.61	OC
**o-cymene**	1022	0.89	0.5	0.32	0.78	0.12	8.45	-	0.4	MH
**β-ocimene**	1044	5.83	1.64	1.27	8.19	0.32	9.9	0.74	1.07	MH
**allyl hexanoate**	1079	2.81	3.36	1.02	0.85	1.03	0.3	4.58	0.5	ES
**nonanal**	1100	4.62	7.65	2.44	1.44	3.29	4.43	4.1	0.41	OC
**octanoic acid**	1167	1.04	0.67	0.12	0.18	-	tr	0.46	1.44	OC
**p-menth-1-en-7-al**	1273	11.06	20.38	1.95	0.77	0.91	1.11	10.3	0.86	OM
**perilla alcohol**	1294	3.85	1.97	6.64	0.83	12.46	9.66	2.6	0.26	OM
**2,4-decadienal**	1315	17.53	11.37	54.59	0.71	13.32	12.13	38.79	1.41	OC
**(Z)-caryophyllene**	1408	1.32	0.45	1.97	0.3	2.11	0.59	0.45	0.26	SH
**geranyl acetone**	1453	4.06	-	-	22.53	-	0.41	0.8	1.66	OC
**pentadecane**	1500	4.32	3.39	-	5.05	-	0.57	2.18	1.01	AH
**ethyl dodecanoate**	1594	1.16	0.34	0.36	0.24	-	0.13	0.12	1.52	ES
**khusimone**	1604	3.37	1.23	3.82	1.68	17.6	10.04	0.69	0.52	OC
**allo-aromadendrene epoxide**	1639	2.42	1.86	2.67	0.66	4.84	3.98	0.59	0.36	OS
**tetradecanol**	1671	-	6.42	-	-	4.3	0.83	3.34	-	OC
**longiborneol acetate**	1684	-	-	-	1.26	-	1.22	-	-	ES
**β-bisabolenal**	1768	3.52	-	-	15.16	-	0.18	0.26	1.45	OS
**octadecane**	1800	4.35	6.11	1.55	2.16	0.98	1.04	1.23	0.38	AH
**nonadecane**	1900	-	1.73	-	4.04	1.15	0.25	0.29	1.61	AH
**Monoterpene Content**		21.64	24.49	10.18	10.57	13.81	29.12	13.65	2.59	
**Sesquiterpene Content**		7.26	2.31	4.65	16.12	6.95	4.75	1.29	2.07	
**Alkane Hydrocarbon Content**		12.54	15.22	3.42	18.78	3.12	2.82	5.12	13.38	
**Ester Content**		17.98	15	6.27	4.21	3.22	4.78	10.67	23.27	
**Other Compound Content**		40.58	42.98	75.48	50.32	72.85	58.49	69.27	58.7	
**Total Content**		99.97	99.99	99.97	99.99	99.95	99.97	99.99	99.99	

Values denote averaged peak area %; RI*—retention indices were calculated experimentally based on retention time of C7-C40 saturated alkane mix Sigma Cat#49452-U and performed spectral matching with a cut-off of 80%, and calculated retention index tolerance of 50 to the Essential Oils GC/MS library.——not detected; tr–occurred in <0.05. Column eleven shows the type of molecule analyzed in the corresponding row; MH–monoterpene hydrocarbon, OM–oxygenated monoterpene, SH–sesquiterpene hydrocarbon, OS–oxygenated sesquiterpene, AH–alkane hydrocarbon, OC–other compound.

### Tick repellency

**Fig 5:**
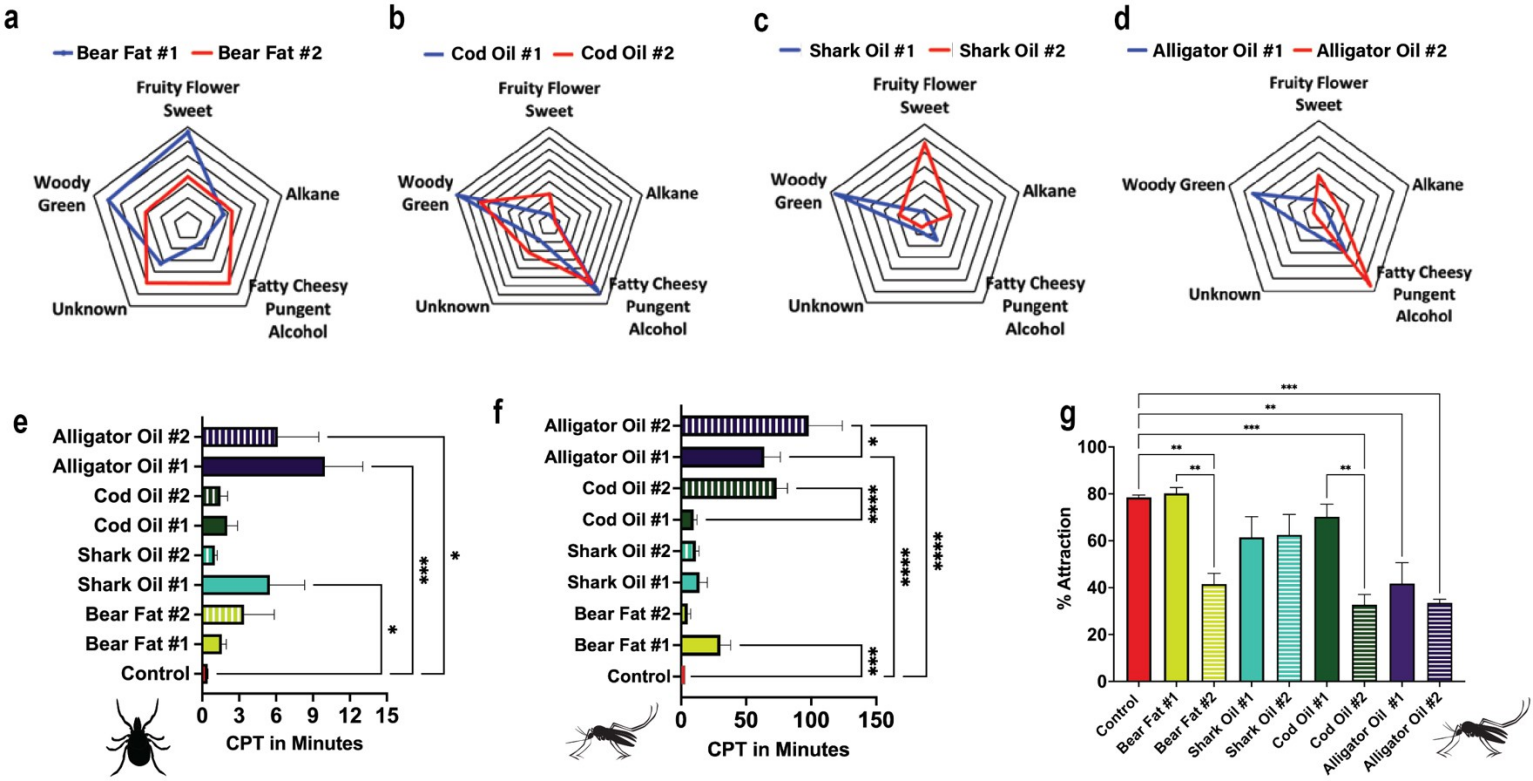
Results of tick and mosquito repellency and odor categorization of animal fats. Shown are the odor categorization of each sample (a-d), and the three behavioral assays conducted (e-g). **a-d. Radar odorant charts.** Odorant categories of each putatively identified compound were weighted based on the summed peak area. Presented in the charts are the relative abundance changes within each odorant category within each fat source and its sensorially more rancid version. Major gridlines represent a 5% change in the summed relative peak abundances of all components contributing to the odorant category in charts A & B and a 10% change in charts C & D. **e. Tick-crawling assay results.** The complete protection time from *I*. *scapularis* tick crossings for each of the different samples tested. The x-axis displays the complete protection time (CPT) in minutes from tick crossing and the y-axis represents the different treatments tested. A Kruskal-Wallis test was conducted to determine the difference between treatments. The resulting significant difference between groups are represented by asterisks (*: p ≤ 0.05 and ***: p ≤ 0.001). **f. Arm-in-cage assay experimental results.** The complete protection time from *A*. *aegypti* mosquito bites was measured for each sample. The x-axis displays the complete protection time (CPT) in minutes and the y-axis represents the different treatments tested. A one-way ANOVA test was used to analyze significance between treatments. Significant differences between groups is indicated with asterisks (*: p ≤ 0.05, ***: p ≤ 0.001 and ****: p ≤ 0.0001). **g. Y-tube olfactometer assay results.** The average percent attraction of *A*. *aegypti* mosquitos to volunteers anointed with different animal fats was measured for each sample. Lower values of % attraction identify a reduction in attraction. The x-axis represents each sample tested, and the y-axis represents percent attraction of mosquitoes to a host treated with a sample or untreated as a control. A one-way ANOVA test was performed to determine the statistical significance of different experimental groups. Significant differences are indicated by asterisks (*: p ≤ 0.05 and ** p ≤ 0.01).

As shown in **Fig 5E**, we found that Alligator Oil #1 conveyed the longest protection time of only 10 minutes, followed by Alligator Oil #2 for 6 minutes, and Shark Oil #1 for 5.5 minutes. All other samples tested did not provide protection from *I*. *scapularis* tick crossings.

### Mosquito contact-repellency analysis

**Fig 6:**
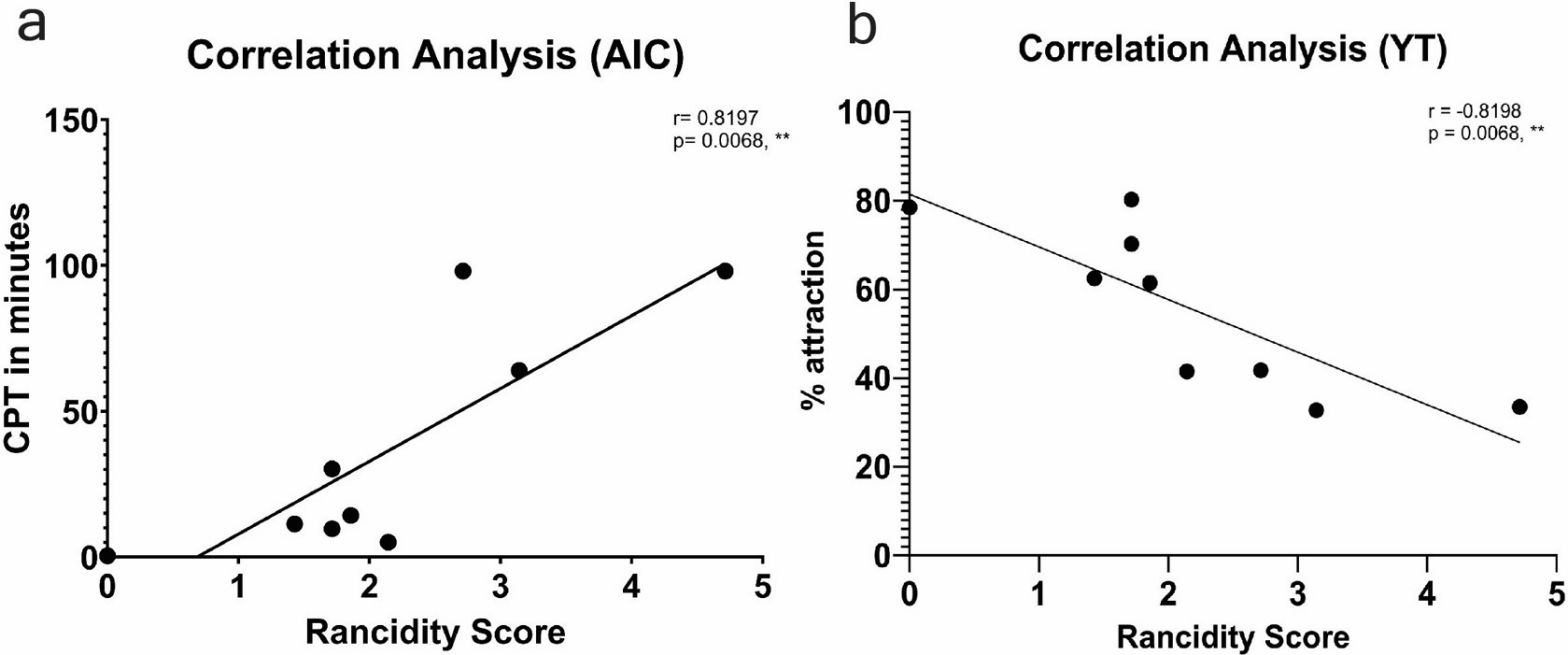
Correlation analysis for rancidity score and contact and spatial mosquito repellency. Shown are the correlation analyses for rancidity score and mosquito repellency. Each point represents a treatment. The Pearson correlation coefficient is represented by “r”. **a. Correlation analysis for rancidity score and CPT.** “AIC” represents Arm-in-cage. The average rancidity score is on the x-axis, and the average complete protection time (CPT) in minutes is on the y-axis. **b. Correlation analysis for rancidity score and percent attraction.** “YT” represents Y-tube. The average rancidity score is on the x-axis, and the average percent attraction is on the y-axis.

**Fig 5F** shows that of the fats tested, Cod Oil #2, Alligator Oil #1, and Alligator Oil #2 provided the longest protection times against *A*. *aegypti* bites with a CPT of 1 hour 13 minutes, 1 hour 4 minutes, and 1 hour 38 minutes, respectively. When comparing fresh and rancid versions of each fat, we found that Alligator Oil #2 and Cod Oil #2 provided significantly longer contact repellency than their less rancid counterparts. Overall, Alligator Oil #2 provided the longest protection time when compared to all the other fresh and rancid animal fat samples. Bear Fat #1 provided longer protection from mosquito bites than Bear Fat #2 with a complete protection time of 30 minutes and 5 minutes, respectively. In a correlation analysis, we found a positive correlation between the rancidity score and the CPT of each treatment (see **Fig 6A**).

### Mosquito spatial-repellency analysis

**Fig 5G** shows that Bear Fat #2, Cod Oil #2, Alligator Oil #1, and Alligator Oil #2 significantly reduced *A*. *aegypti* mosquito attraction compared to both the untreated control and their fresh versions. When comparing Alligator Oil #1 and Alligator Oil #2, both treatments conveyed a similar reduction in attraction. Shark Oil #1, Shark Oil #2, Bear Fat #1, and Cod Oil #1 did not reduce the attraction rate of mosquitoes in the presence of a human bait. In a correlation analysis, we found a negative relationship between the rancidity score and the percent attraction of mosquitoes (see **Fig 6B**).

## Discussion

Ethnohistorical accounts suggest that indigenous peoples of the Gulf Coast region used animal fats to repel mosquitos. The strong scents mentioned in these accounts suggest that these fats were rancid (see **Table 1**). To our knowledge, neither the use of animal fats as mosquito repellents, nor the impact of rancidification of such fats on their repellent potency have been explored scientifically. To fill this knowledge gap, we conducted bioassays to measure the spatial and contact repellency properties of fats from different animal sources, that varied in rancidity. In our study, we used the Yellow fever mosquito *Aedes aegypti* as a model organism to test mosquito repellency of different animal fats at different states of rancidification. This particular species is a anthropophilic human biter recommended by the World Health Organization to test mosquito repellents [42].

A study from 2002, found that human olfaction can be used as a tool to accurately predict levels of rancidity in oils [43]. Our GC/MS analysis clearly shows that the terpene profiles vary between the different fat samples we tested (**Fig 4**) and the rancidity scores that we determined via human olfaction, were effective predictors for mosquito repellency. The fats that had the highest rancidity scores outperformed the less rancid animal fats both as spatial and contact repellents. For example, the rancid smelling cod liver oil (Cod Oil #2) significantly repelled mosquitoes while the less rancid Cod Oil #1 did not. Our results suggest that rancid animal fats are more effective mosquito repellents than fresh animal fats, this is validated by our correlation analyses (**Fig 6**). We found a positive relationship between how rancid a treatment was and how long that treatment protected from mosquito bites. Inversely, we found a negative relationship between how rancid a treatment was and the percent mosquito attraction to a human. This trend was not seen with ticks since *I*. *scapularis* ticks were not repelled by any of the fats tested in this study. Animal fats have long been employed by Native Americans for functional and cultural purposes [44]. Animal fats were used for culinary purposes such as cooking, binding foods (like pemmican), pigments and medicines. Alligator and other animal grease or fat products would likely have served as an all-purpose salve for chapped, burned or wounded skin, a hair pomade and as a dressing for moisture-sensitive tool kit items like bows and bow strings [29]. One account of the Akokisa of the east Houston area suggests the substance also served as a sunblock, lamp fuel and a floatation-enhancing rub [45].

One cannot rule out the possibility that animal fats were combined with some other botanical or mineral substances to achieve a repellency effect. Given that animal fat products were universal binders in many ancestral societies, it seems unlikely that animal fats in grease or oil forms would have been used in a pure state for skin daubing. The Native American Ethnobotany Database lists seven concoctions employed by the Iroquois for mosquitos which combined bear grease and nut oils including hickory, chestnut, hazelnut, beechnut, butternut, and oak [28, 29]. Beyond botanical additives, mineral-based ingredients were probably also combined with animal fats to leverage the added benefit of sunscreen [46]. For instance, the Maori of New Zealand reportedly used bird fat, shark oil, or moa marrow combined with red ochre and other substances as a multifunctional repellent and sunblock [47]. Likewise, the Beothuk of Newfoundland are reported to have combined bear grease and red ochre for anointing, to produce a red-colored armor that protected from insects and the sun [1, 48]. The Ovahimba, hunter-gatherer pastoralists of Namibia, currently use red ochre mixed with clarified butter for many symbolic and functional purposes including sunscreen and as a robust mosquito repellent [46].

Interestingly, experiments by Rifkin [46] testing the repellency of red ochre and animal fat binders among the Ovahimba, showed that dairy-based fats mixed with ochre provided mosquito protection. Ochre and non-dairy animal fats combinations were not found to provide any protection, and concurrently the rancidification of animal lipids were hypothesized to attract mosquitos by producing scents similar to that of humans [46]. The results of our study contradict this observation and rancidity appears to be positively correlated with mosquito repellency. In our Y-tube olfactometer assay, we found that Bear Fat #2 and Cod Oil #2 provide significant reduction in *A*. *aegypti* mosquito attraction compared to their less rancid counterparts. When comparing two rancid versions of alligator oil, Alligator Oil #1 and Alligator Oil #2, both treatments showed a similar spatial repellency. Meanwhile, both shark fats were not observed to be particularly rancid and did not provide significant repellency to *A*. *aegypti* mosquitoes. This data suggests that rancidity may be a key component for repelling mosquitoes using animal fats.

When comparing the volatile chemical composition from fresh and rancid animal fat samples, certain short organic molecules were observed to change in concentration. Mono- and sesquiterpenes are organic secondary metabolites which are volatile in nature. It is particularly the monoterpenoids which confer aroma, taste and bioactivity particularly to essential oils and other natural products [49]. It can be seen from **Table 3**, that there is rise in the total percentage content of these compounds from fresh to rancid, across all four animal fats tested. This is to be expected as the rancid oils were shown to be more pungent in their smell than their fresh counterpart. Alkanes are the least reactive type of hydrocarbon; however, they have some commercial value in the fuel industry and some alkanes act as important chemical messengers for a wide array of organisms including insects [50, 51]. Similarly, these compounds see a rise across all oils from fresh to rancid besides Cod Oil which sees a small but not too significant decrease in total alkane hydrocarbon content. Of the other components present, those that bear importance in terms of rancidity and insect repellency are the carboxylic acids, hexanoic acid and octanoic acid as they are described in the literature to confer a rancid scent to its source and has been dubbed a marker compound for rancidity [52, 53]. As mentioned in the results, Alligator Oil is the only oil in which both of these compounds mutually occur in a notable amount, with the level of hexanoic acid increasing significantly from oil #1 to oil #2 and there being an increase for octanoic acid as the oil becomes more rancid. This is to be expected as the Alligator Oil, had the strongest smell and longest CPT in comparison to the three remaining oils. Ethyl dodecanoate, can be regarded as an important constituent identified. This is due to its recognized deterrence activity towards insects [54]. Both Cod Oil and Alligator Oil see a rise in the area percentage of this ester from fresh to rancid oil while the remaining fats see a decrease. This trend remains true for the total ester content in which again, the area percentage is increased for the rancid type oil whereas with Shark Oil and Bear Fat, the fresh type oil has the greater area percentage than the rancid type. The esters found present to be in the animal fats are considered significant chemical compounds for this study, as they are reported to be responsible for the characteristic scents of many biological products and organisms [54, 55]. Nonal and geranyl acetone are reported chemo-attractants for insects, specifically the *Culex* genus of mosquitoes [56]. An interesting observation is made for Alligator Oil whereby nonal, decreases as the rancidification occurs, perhaps limiting its chemo-attractant activity in the second more rancid oil. The aldehydes, 2,4 heptadienal and 2,4 decadienal are known to be oxidative markers of rancidity in certain oils such as linolenic acid. These compounds form and can be used to measure the progression of lipid oxidation [57]. Interestingly, in the animal fats investigated, these chemical constituents don’t see much difference between fresh and rancid oil. However, in Alligator and Shark Oil their peak area percentage is reduced in oil #2. As can be seen from the table above, those compounds which have known characteristics, such as strong or rancid-like odor and insect repellent bioactivity, see a general rise as an oil oxidizes from fresh to rancid. Fats which had higher CPTs contained those compounds which have reported insect bioactivity at greater concentrations than fats with low CPTs.

One potential explanation as to why animal fats repel *A*. *aegypti* mosquitoes but not *I*. *scapularis* ticks could be due to the different types of sensory receptors that these two types of organisms use to sense olfactory cues. Analyses of their genomes has shown that mosquitoes having odorant receptors, ionotropic receptors, and gustatory receptors while ticks only have ionotropic receptors and gustatory receptors and lack odorant receptors [58–60]. One hypothesis that would explain the difference in sensitivity between ticks and mosquitoes to rancid animal fats is that the active ingredients in these fats interact with odorant receptors. Further research is necessary to clarify this point.

Our findings provide support to ethnohistoric accounts that some of the Karankawa, Akokisa, and Atakapa purposely anointed themselves with rancid alligator fat to repel mosquitos [25]. Nonetheless, these finds do not exclude other purposes for anointing with alligator fat or other animal fats. Both the bear (black bear, *Ursus americanus*) and fish (Cod, *Gadus sp*.) fats obtained for this study, are not biogeographically relevant to the Gulf Coast bend region and may not accurately represent local analogs.

Although many Native American ethnobotanical remedies for biting insects have been recorded, the use of animal products for this purpose is less well known. In this study, we found that rancid animal fats can be developed into effective mosquito repellents. This correlates with the descriptions in ethnohistorical accounts. Traditional knowledge and zooethnological strategies for dealing with mosquitos would have been important among indigenous Gulf Coast peoples for coping with alien diseases like malaria and yellow fever following the Columbian exchange.

An interesting question is how the efficacy of our self-rendered animal fat-based mosquito repellents compares to modern-day repellents that are widely available for purchase over-the-counter in developed countries. The most-used active ingredients are DEET, picaridin, and oil of lemon eucalyptus (PMD). Commercial mosquito repellent products with these active ingredients, when applied to human skin, can produce protection times of up to six hours or longer [35, 61–63]. While this is roughly four times the protection time we measured for the best-performing animal fat-based repellents, it must be noted that we only applied a thin layer of fat on the volunteer’s skin. Future studies may address the question if using larger amounts and thicker layers of rancid fats could prolong its insect repellent effects.

## Supporting information

S1 File Contains S1-S4 Tables showing raw data of all experimental assays conducted and the historical context statement. (DOCX)
