# Supplementary material for: Rancid rumors or Native wisdom: Evaluating the efficacy of animal fats as insect repellents attributed to historic-period Native Americans
Source: PLoS One. 2024 Jul 17;19(7):e0301677. doi: 10.1371/journal.pone.0301677 (PMC11253976; doi:10.1371/journal.pone.0301677)
Supplement: S1 File — (DOCX) [file pone.0301677.s001.docx]

**Raw Data**

**Supplemental Table 1.** Arm-in-cage assay raw data.

**b**

**a**

**c**

**d**

**e**

**f**

**h**

**g**

**Supplemental Table 2.** Tick skin-crawling assay raw data.

**b**

**a**

**d**

**c**

**f**

**e**

**g**

**h**

**Supplemental Table 3.** Y-tube olfactometer assay raw data.

**a**

**h**

**g**

**f**

**e**

**d**

**c**

**b**

**Supplemental Table 4.** Putative identifications of Odorants by GC/MS


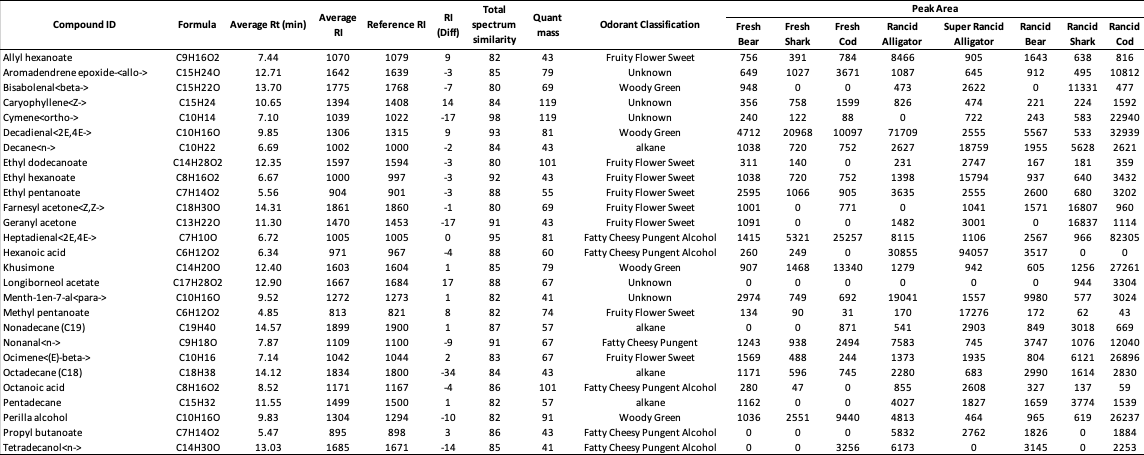


**Historical Context**

The written record of mosquitos in the Gulf Coast area begins with the earliest Spanish entradas ca. 1520-1540 and continues through the 16^th^ century wanderings of the La Salle Expedition. During this time malaria (*P. falciparum*) was endemic among Europeans and individuals taking part in these expeditions would have easily transmitted this disease to endemic New World *Anopheles* mosquitos and indigenous American peoples. The timing of this transfer is likely mosaic in nature but the outcome is certain. European forces introduced alien malaria and yellow fever carrying *Anopheles* and *Aedes* mosquitos to the western hemisphere. These diseases became among the most prevalent killers in the North American colonies in the 16^th^ and 17^th^ centuries ^1^.

While introduced diseases such as smallpox among American Indians is well documented, the impacts of mosquito vectored disease is scarcely considered. Schaeffer ^42^ conducted a survey of skeletal lesions associated with known malaria cases among historic Native American individuals. Most malaria-like lesions occurred in protohistoric (AD 1492-1686) populations. These findings suggest that malaria and European mosquitos may have had an underappreciated impact on indigenous Americans, much of which may have occurred prior to sustained contact.

Mosquitos are documented in nearly every written account of European travelers in the Americas. However, comments on repellent anointing behaviors do not enter the written record until the mid 18^th^ century. Given that Europeans were struck by the scent of these seasoned animal fat repellents, it seems unlikely that they would have gone unnoticed in the narratives of protohistoric period travelers. If rancid animal fat repellents emerged only after sustained contact, they might reflect an innovative adaptive response to invasive mosquito transmitted diseases in the Gulf Coast area.
